# The Role of Physical Activity in Cancer Recovery: An Exercise Practitioner’s Perspective

**DOI:** 10.3390/ijerph19063600

**Published:** 2022-03-18

**Authors:** Clare M. P. Roscoe, Andy Pringle, Charlotte Chandler, Mark A. Faghy, Ben Barratt

**Affiliations:** Human Sciences Research Centre, University of Derby, Derby DE22 1GB, UK; a.pringle@derby.ac.uk (A.P.); c.chandler@derby.ac.uk (C.C.); m.faghy@derby.ac.uk (M.A.F.); ben.barratt1@gmail.com (B.B.)

**Keywords:** cancer recovery, physical activity, exercise practitioners, health care professionals

## Abstract

Less than 20% of cancer patients meet the recommended physical activity (PA) guidelines, partially due to poor knowledge and enforcement/encouragement amongst health-care professionals (HCPs). The primary aim of this study was to explore the perceptions of exercise practitioners on the role of PA and the physiological and psychological benefits to recovering cancer patients; the secondary aim was to understand the barriers and facilitators of promoting PA to cancer survivors. The third aim was to, seek the perspectives on the effectiveness of referral systems between the hospitals and PA structures. A purposive sample of five exercise practitioners’ (four male and one female) with experience with cancer patients participated in a semi-structured interview (45–60 min). Interviews addressed five key topics: intervention procedures, patient well-being, patient education on PA, effectiveness of referrals from hospitals, and post-intervention PA. Interviews were transcribed verbatim and analysed via thematic analysis. The participants believed that recovering cancer patients possess a knowledge of the physiological benefits of PA, yet psychological understanding remains unknown. Social environments are key to participation in PA and most HCPs lacked knowledge/awareness of the benefits of engaging in PA. There is a need to improve HCPs knowledge of the benefits of PA, whilst providing standardised training on how PA can improve cancer patients’ outcomes.

## 1. Introduction

From 2016–2018, there were approximately 375,400 new cases of cancer in the United Kingdom every year, which is equivalent to 1000 every day [1], with 53% of these being either breast, bowel or prostate cancer [2]. This number is expected to rise to four million by 2030 [3] and 5.3 million by 2040 [4]. As a result, an associated increase in the number of individuals undergoing invasive treatments to tackle cancer, such as surgery, chemotherapy and radiation therapy were observed [5]. Physical activity (PA) can be an effective way to help improve survival rates, as research shows that it leads to physiological improvements such as increased flexibility, haemoglobin levels and bone mineral density [6]. However, there is evidence to suggest that 20% of cancer patients who have undergone treatment are not meeting the daily PA guidelines of 30 min of moderate PA five times per week [2]. This statistic exists despite the knowledge that PA can alleviate both physical and psychological symptoms associated with cancer treatments, such as chronic fatigue and self-efficacy, and ultimately improve quality of life [7].

Routine cancer treatments are associated with several side effects, the most common being post-cancer fatigue (PCF), which affects between 70 and 100% of patients during and post treatment [8]. PCF leads to side effects such as muscular atrophy, weight loss, reduced aerobic capacity, increased social anxiety and depression, contributing to reduced quality of life and engagement with PA [8]. Typically, cancer patients would be advised by their doctors to ‘rest and reduce their physical activity’; however, if the patient experiences no pain or shortness of breath, then PA is now recommended for physical benefits and quality of life [9]. Subsequently, the American College of Sports Medicine [10] now recommends that recovering cancer patients participate in a minimum of 150 min of moderate-intensity aerobic PA per week, such as brisk walking or swimming. However, studies suggest that most cancer survivors are insufficiently active, with those meeting the PA guidelines ranging from 17% to 47%, which results in a significant increase in the risk of developing secondary cancer or chronic illness [11,12,13,14,15,16,17,18].

Despite the benefits of PA being recognised by recovering cancer patients, many cancer survivors do not adhere to clinically advised levels, as stated previously. The impact of this is that the symptoms of the cancer and side effects from the treatment will cause physical, psychophysical and psychological deterioration [19]. Research exploring patient perspectives via the use of questionnaires and focus groups have highlighted several barriers to PA for cancer patients, for example, the inability to set achievable goals resulting in lowered motivation compared to pre-cancer status [20], and a lack of PA facilities and readily available information [21]. Furthermore, Knowlton et al. [22] found that patients believed the burden of their illness prevented them from undertaking PA, as their body would not let them take part. These studies highlight the need for cancer patients to receive education on PA and the benefits it can provide to help them improve their quality of life. There is evidence that social support can play an important part in PA adherence [23], and it is therefore important to consider the role that health care practitioners (HCP’s) can play in supporting cancer patients to become more physically active as part of their recovery. 

In the UK, a wide range of HCP’s support cancer patients throughout their recovery, including GPs, oncologists, surgeons, nurses and health-care assistants. It is vital that HCPs possess a standardised knowledge of PA and how it can benefit cancer patients, as some HCPs do not prescribe PA for cancer recovery [24], which may be due to a lack of knowledge regarding the benefits of PA [25]. Additionally, programmes such as Cancer and Rehabilitation Exercise (CARE) have had variability and inconsistencies in how people have discovered their service, with some HCPs routinely promoting the service, some through patients conducting their own research and others word of mouth; this highlights the requirement for more effective and routine promotion to the CARE programme [23]. With this in mind, exercise rehabilitation programmes have been implemented across the UK for recovering cancer patients, aiming to reduce the severity of PCF [26]; these are typically delivered in a local public leisure centre, by a trained cancer rehabilitation practitioner. That said, there are significant variations and inequalities in rehabilitation services that are provided for individuals living with and beyond cancer [27], making it difficult to standardise, define and measure approaches that are effective (settings, intensity), which therefore makes this a major challenge [28]. Research has shown oncologist recommendations to be the most influential with regards to cancer patients’ motivation to undertake PA [29]; therefore, it is essential that oncologists are aware of the benefits of PA and are confident recommending PA for cancer recovery.

Research has shown that PA helps alleviate the symptoms of cancer-related fatigue by increasing VO_2_ max results (improving aerobic performance) [30] and increasing flexibility, thus reducing physical pain [31]. Van Vulpen et al. [32] conducted a meta-analysis which found that supervised PA sessions elicited significantly more improvements in fatigue reduction compared to unsupervised sessions, highlighting the importance of controlled rehabilitation programmes. There is also a dearth of literature highlighting the psychological benefits of participating in these PA interventions. Segar et al. [33] found that a 10-week moderate aerobic exercise intervention for recovering breast cancer patients reduced their symptoms of depression and state/trait anxiety, whilst Courneya et al. [34] found an increase in patient self-esteem and self-confidence in a similar exercise intervention-oriented study. This, therefore, suggests that engagement in exercise interventions is important for a healthy recovery, as well as an improvement in quality of life. Further research has explored the need to encourage recovering cancer patients to continue exercising post-intervention. Queen, Bloxham and Brow [35] found that for patients to continue being physically active without the assistance of internal systems such as practitioners or physiotherapists, they must have a close, supportive external system such as close partners/spouses to keep them motivated, with self-confidence being a vital factor in cancer patients’ PA participation [36]. To summarise, and based on the reported research, it is essential to engage HCPs in the promotion and delivery of PA as identified in key government strategies [37], as this is imperative for individuals living with cancer to ensure that they receive important information which can shape, design and deliver PA to them. With these thoughts in mind, this research aims to explore the perceptions of cancer rehabilitation exercise practitioners on the role of PA, with the objectives of understanding the physiological and psychological benefits to recovering patients, thoughts on the barriers and facilitators of promoting PA with cancer survivors, and finally understanding the effectiveness of referral systems between the hospitals and PA structures.

## 2. Materials and Methods

### 2.1. Participants

A purposive sample of five cancer rehabilitation exercise practitioners (four male and one female) consented to be interviewed. All participants were specifically trained in PA promotion to cancer patients, and they actively worked and promoted PA to cancer survivors at the time of the research. These specific HCPs were considered significantly involved and knowledgeable in PA promotion and were therefore selected for the study. The participants were based in a variety of settings, for example, in local leisure centres and employed on a community basis. Participants’ country of work varied across different continents; the United Kingdom (England, Midlands and Scotland, Glasgow), Canada (Toronto) and Australia (Perth). The participants had all worked in their fields from six months to 30 years, ensuring sufficient knowledge and experience. 

### 2.2. Instrumentation

Data was collected via semi-structured, one-to-one interviews, which have been identified as effective in providing in-depth and insightful accounts from health professionals [38], including those in PA/cancer interventions. Five main topic areas were identified prior to the interviews which addressed a wide variety of PA experience and its implications. These were: (1) the effectiveness of PA interventions to include barriers and facilitators surrounding PA; (2) patient well-being addressing physiological and psychological benefits; (3) patient education on PA; (4) the effectiveness of referrals between hospitals and PA structures; and (5) post-intervention PA. These topics were developed from Bennett et al. [39] and Sutton et al. [40] and covered/investigated exercise practitioners perspectives on PA knowledge and provision of cancer survivors. The questions asked were predominantly open ended, as these allow for the exploration of topics in depth, the identification of processes, and also for the recognizing of possible causes of observed correlations [41]. 

### 2.3. Procedure

All subjects gave their informed consent for inclusion before they participated in the study. The study was conducted in accordance with the Declaration of Helsinki, and the protocol was approved by the Ethics Committee of the University of Derby (30 November 2018, ETH011118). Following University ethical approval, purposive sampling involved the principal investigator Ben Barratt emailing the participants directly to recruit participants. Interviews took place in person and online via Skype, and they lasted between 45–60 min and were terminated at the point where the discussion naturally ended.

### 2.4. Data Analysis

Each interview, be that face-to-face or online, was digitally recorded and transcribed verbatim, reviewed for grammatical accuracy and re-read for familiarity by the lead researcher (Ben Barratt). Data was then analysed through a process of thematic analysis, guided by Braun and Clarke’s [42] six-stage process (familiarising oneself with the data, generating initial codes, searching for themes, reviewing themes, defining and naming themes, and producing the report) and acknowledging that thematic analysis is flexible/reflexive [43]. Following transcription and immersion of the interview transcripts to saturation, the scripts were read and re-read for familiarisation and to check for accuracy, and then coded to address stages one and two. Codes were further grouped into sub-themes and themes identified via repetition in topics; similarities and differences in answers to a question; a reflection of missing data in the research; theory relating to the scientific underpinning of the questions; and, finally, the metaphors and analogies participants used when answering questions [44]. To facilitate trustworthiness and credibility, the second author reviewed the transcripts in order to reduce the potential for researcher bias [45]; any conflicts, albeit minimal, were discussed and final themes were agreed between the first and second authors. Yardley’s [46] principles were adhered to during data analysis, which consisted of conducting a thorough analysis of the raw data, ensuring quotes identified related to the themes and aims of the study, and acknowledging the interpretation of the findings. The authors agreed to not utilise member checking of the transcripts, as there is little evidence that this increases the trustworthiness of the data [47]; similarly, this process asks for more commitment from the participants which was considered an unnecessary request on their time.

## 3. Results

Following analysis, four themes emerged: (1) the importance of social support for cancer patients; (2) cancer patients’ engagement with PA and the factors that influence this; (3) HCP’s knowledge and provision of PA prescription for cancer patients; and (4) the impact of policy and Government support on service provision. To protect anonymity, participants were referred to by P and a number.

### 3.1. Importance of Social Support for Cancer Patients

Participants highlighted the importance of social support for recovering cancer patients from what they had witnessed, particularly in relation to their engagement with PA. Practitioners discussed the benefits of group PA modes in facilitating involvement from like-minded individuals. P4 highlighted that “it’s this social support element that will keep [patients] involved in PA as they are enjoying themselves”. In some cases, the practitioners likened the social support between cancer patients to that of a family, stating that “as they are all in similar situations, they bounce off each other and it almost becomes like a family which makes them look forward to exercising each week” (P3). The likening of this social support to the notion of family implies a strength of relationship beyond that of usual friendship, reflecting the challenges the cancer patients faced. P1 referred to this challenge by stating that “the patients are all with like-minded individuals; they’ve all been through their very own respectable level of trauma in their lives” suggesting that cancer patients’ shared trauma can contribute to the bonds they create with one another. 

The importance of this shared experience was further discussed in relation to group PA, which provided a form of psychological support for the patients. P2 acknowledged that some cancer patients may experience anxiety or depression because of their illness, yet having “gone through the same thing”, their relationships were strengthened, suggesting that shared experiences provide an important source of empathy. The uniqueness of these relationships was also acknowledged; P1 referred to one cancer patient who said that “the support within the programmes helped to facilitate a social network and group of friends that couldn’t be replaced anywhere else”. This reflects the strength of programmes in facilitating a social network that enables cancer patients to bond and is something that should be promoted in all cancer rehabilitation programmes alongside the formalised PA sessions. Significantly, the unique relationships this cancer patient developed were influential in encouraging them to maintain engagement in the programme, even when this meant self-funding. This further highlights the value of social support for continued engagement in PA. The exercise practitioners identified the importance of social support and long-term engagement in PA. As P5 stated, “I have some patients who were diagnosed over ten years ago and are still attending the group-based sessions because of how many friends they have made and how much they enjoy the social element”. Social support is therefore highlighted as both a determinant and consequence of engaging in these group sessions. 

An important element of social support appears to be how those new to the programme are supported by experienced attendees. P5 discussed how “a newly diagnosed patient may be worried about their treatment, [or] an upcoming review/assessment, and one of the more experienced patients would say ‘I used to be like you but I’m doing great now’. So, this social element really does inspire patients”. Newer cancer patients appear to learn vicariously from their more experienced counterparts and, as a result, increase their self-efficacy in PA. All practitioners recognised that despite its benefits, some aspects of group PA were not suitable for every individual patient and some individuals prefer to participate in PA alone, P2 provided a specific example of why this may be the case:

*“[The social aspect] doesn’t work for everyone because, for example, a lady lost her hair and did not want to be in large groups doing exercise because they were conscious [of] how they look. So, in this case we offer home exercise plans, use of a pedometer etc…basically we try to strip down all the barriers so we can get them more active”.*

It is important to balance the benefits of social support with the need for PA to be tailored to the individual in overcoming personal barriers. For example, P4 described cancer patients who “don’t want to be recognised as a cancer survivor, they just want to go [to] their own gym and do their own thing”. Some individuals may not wish to identify as a cancer survivor or have others perceive them in this way, and instructors need to be aware of this preference. Therefore, the support exercise practitioners offer may be less tangible in the form of a structured programme, and instead offer the patient freedom to dictate their own PA in accordance with their self-perception and individual needs. These thoughts are important in relation to the study’s aims and objectives, as it is imperative to consider social support in terms of a facilitator for PA while being wary that some cancer patients do not want to be considered as a cancer survivor and are happy to be an individual in their recovery and PA participation post cancer. 

### 3.2. Cancer Patients’ Engagement with Physical Activity and Influencing Factors

The exercise practitioners discussed cancer patients’ engagement with PA in terms of their knowledge of its benefits and their concerns about PA, given their illness. The exercise practitioners perceived cancer patients’ knowledge of the benefits of PA to be varied; for instance, P1 considered younger patients to have a better general understanding than older patients. However, the practitioners had experienced different levels of knowledge amongst cancer patients, particularly in relation to wider, non-physical benefits. When asked whether cancer patients were aware of the psychological benefits of PA, P5 stated “I would say initially not, a lot of them are just there to get their energy back, but then they realise that they have gained such a boost and feel so much better”. Conversely, P1 felt that their patients have “an incredibly strong knowledge of the psychological and social benefits”. Knowledge and perceptions will inevitably vary with the patient populations. Although not their initial motivation, the cancer patients will often become aware of the wider psychological and social benefits of PA as they progress, suggesting that their knowledge and interest incubates through the programme. It therefore appears important to ensure that cancer patients are aware of the mental and social benefits of PA at the point of referral to a programme, and to act as a facilitator for their engagement.

Understandably, the practitioners were advocates for PA as part of a patient’s cancer recovery, based on both their own knowledge and what they had witnessed when delivering programmes:

*“Every person that I have met with cancer, exercise has had a positive effect on their lives in some way, whether that be a physical, mental or social benefit such as cardiorespiratory fitness, reduced anxiety or enjoying themselves and making new friends who understand what they are going through”.*

(P5)

Despite the known and perceived benefits of PA, practitioners reported that cancer patients lacked knowledge about how and when to be active. A significant barrier to patients’ engagement with PA appears to be concerned with whether it was safe for them. P2 highlighted an example of misconceptions related to PA:

*“There is a notion out there that rest is the best thing for recovering from cancer, as the last thing you would think to do when recovering is to go out and exercise… people think that it would put their bodies under further harm, which is obviously not the case at all if done correctly”.*

This was confirmed by P1, who stated that one of the most significant barriers to PA is patients’ negative perceptions, as they are worried about fatigue and doubt that PA can relieve this. Practitioners felt that cancer patients were more inclined to align their recovery with previous advice relating to rest, rather than the developing evidence that PA should be prescribed. This was highlighted by P3, who stated that “[patients] are unaware that it’s safe for them to exercise whilst undergoing/finishing treatment…it stems back to years ago where the thought process was ‘rest is best’, but I think we’re moving away from that now”. The practitioners acknowledged that changes to PA prescription for cancer recovery are emerging, highlighting that “as of about five years ago, exercise was often under-dosed because we were so afraid of how it would affect patients...and we were afraid of pushing them” (P4). While it is important that PA recommendations are made with a patient’s safety in mind, the developing evidence is that PA is safe and beneficial for cancer recovery, and patient education in relation to this information would therefore appear beneficial.

The exercise practitioners also highlighted that cancer patients lacked knowledge in terms of the wider psychological benefits of PA, and they identified this as a barrier. This was represented by fears related to their physical capability, as P1 stated that cancer patients “feel that their fatigue creates an atmosphere where they might not be able to achieve, and failure is inevitable”. P4 stated that, aside from their illness, patients will begin to recognise that PA generally makes them feel better, but also that “patients don’t understand the magnitude of how much [the] psychological benefits [of PA] impacts quality of life due to the reduction in anxiety levels [and] depression”. A cyclical relationship between engagement in PA and a cancer patient’s mindset is apparent, as their belief in their physical capabilities acts as both a barrier to, but also improves because of, PA. 

To help increase cancer patients’ PA engagement, one of the strategies discussed by all practitioners was goal setting. P1 highlighted that “the motivational benefits of achieving goals every single day helps massively with reducing levels of…depression and anxiety”. P5 discussed how goal setting was incorporated into the first session they have with a cancer patient, to understand them more broadly as a person and more specifically their aims for the programme. They described this first session as the ‘most important’ and that motivational interviewing and behaviour change are a key focus in understanding how they can best offer support. It is important that the PA practitioner is involved in the goal setting process to ensure that the goals patients set are realistic for, as P5 stated, “some patients are over ambitious, and some are under-ambitious”. Practitioners also emphasised the importance of ensuring that goals are tailored to the individual and relate to both PA and daily life. P1 provided an example goal of walking an extra lap around a supermarket, describing this as “innocuous to us but…huge for the patients”. Practitioners therefore highlighted that the nature and difficulty of the goals set should be scaled according to the individual and their capabilities, recognising that simple everyday tasks may now be more difficult, but that they also provide the opportunity to increase PA. 

### 3.3. Health Care Practitioner’s Knowledge and Provision of PA Prescription for Cancer Patients

The third theme evidenced within the data relates to how PA as a treatment is delivered to cancer patients and encompasses aspects relating to the prescription of PA and referral onto relevant programmes. A key topic of discussion from the exercise practitioner’s perspective was the knowledge of HCPs and the degree to which this could affect their referrals to PA programmes. HCPs knowledge was considered in relation to the physiological benefits of PA for cancer recovery, with P5 highlighting that “when I give a talk to [HCPs] and show the…effects on hypoxia, reduction in fractures, gene expression et cetera, they’re not aware of these clinical effects”. Both P1 and P2 highlighted that this lack of specific knowledge may affect an HCP’s likelihood of prescribing PA, with P1 stating that HCPs are “extremely knowledgeable about the benefits, but in terms of applying that to an exercise class, prescription it’s not done as frequently as maybe it should”. These quotes suggest that although HCPs possess a wealth of medical knowledge, they may not be aware of specific PA benefits for cancer patients and therefore are less inclined to prescribe PA. P3 also highlighted the variability of HCPs knowledge and referral practice, stating that “Some [HCPs] are absolutely amazing and are real drivers for exercise and will encourage their patients to exercise and join programmes…yet… some only have a vague awareness of exercise and don’t fully understand it’s true benefits [for] patients”.

Exercise practitioners provided their perspective of HCPs knowledge in relation to their awareness of PA programmes available to cancer patients. The consensus across exercise practitioners was that not all HCPs were sufficiently aware of the number or location of PA programmes on offer, with P4 highlighting that this may be due to PA for cancer recovery being a relatively new field and therefore something HCPs may not be informed about. P3 provided a recommendation for how this could improve:

*“The HCPs need to be more aware of the local exercise programmes... not only do the oncologists need to know about this, but I think it’s important that the whole team should be aware of the programmes. For example, when the patients meet with the cancer nurse specialist who completes a holistic needs assessment… so it would be wise for them to also know about the local programmes”.*

Awareness of PA programmes for all HCP’s involved in a patient’s cancer treatment could increase the likelihood that PA will be prescribed, allowing for recommendations at varying stages in the treatment and recovery process. The frequent recommendations for PA can increase patient awareness, as highlighted by P5 who suggested that HCPs “need to bring up talking about PA more often with the patient so that it can be in their mind that exercise is an option for them”. This would ensure the enhanced communication of the programmes available, as opposed to cancer patients learning about them by chance, as P2 stated that “a patient may literally just see one of our posters in the hospital and ring us up to get more information and see if they are eligible to join the programme”. 

The exercise practitioners appreciated that a lack of knowledge and therefore referral may be related to resources, given that HCPs may have limited time with each cancer patient, and that a PA prescription may therefore not be a priority. The issue of resources in relation to HCPs time and the ease with which they can refer was discussed, and the data suggests that improvements could be made in this regard. P3 discussed the issue of time, stating that:

*“When [a] HCP has a meeting with a patient, they are not long conversations and speaking about exercise may not be on their radar…spending 10–20% of their time speaking about exercise may then result in them not being able to speak about other important things when it comes to their recovery”.*

Understandably, a consultation between an HCP and cancer patient must focus on their medical treatment and recovery, which may not leave time for discussions related to PA. This highlights the importance of the wider team of HCP’s having PA knowledge and the confidence to promote it. P1 described access to PA as a barrier, as there are “a lack of resources and knowledge [regarding] how to go about getting involved in exercise, [so HCP’s] ultimately end up disregarding the idea of exercising”, even if the HCP intends to recommend PA as a treatment. P4 stated that “there is no centralised database that an [HCP] can log on to and see a list of local [exercise] practitioners in the area”, and it therefore appears necessary to improve HCP’s access to information about relevant, local PA initiatives to improve the ease with which they can refer a cancer patient. The practitioners offered some recommendations regarding how the links between HCPs and PA programmes could be improved, with P5 offering a positive example of a “physio whose job is to do a consultation with the patients and those who are suitable are then referred straight into a programme”. P1 added a recommendation:

*“The oncologist [could] actually phone me during a meeting with the patient or even me being there in person…it would give us that immediate prescription and the patient would know straight away who I am, what I represent and how I can help them”.*

This suggests that should exercise practitioners be involved in a consultation between HCP’s and cancer patients, a prescription of PA and referral to a programme could occur sooner. However, it may be difficult for the HCP and exercise practitioner to agree on what is best for the individual patient, as P2 stated:

*“We attended a health and well-being meeting [with] the HCP’s …and a cancer nurse specialist said that they had a patient who had just undergone breast surgery and wasn’t suitable for referral at this particular time. But in our eyes, that’s our decision to make because we could have a consultation with her, and she could be fine to begin exercise”.*

This may highlight HCPs lack of awareness of the benefits of PA for cancer recovery; however, it also addresses the need for all parties to work collaboratively. There are also perceived challenges with regards to the actual referral mechanisms from HCP to PA programmes. The need to develop a quick and easy process for HCPs to refer a cancer patient was highlighted by all exercise practitioners, as healthcare and PA provision are “two complete separate sectors in a way…[so] trying to establish a smooth route with referrals is often difficult” (P3). Exercise practitioners’ involvement in HCP-patient consultations could help speed the referral process up, which P2 has experienced:

*“[I’m] involved in the monthly cancer nurse specialist meeting… so all the HCPs will meet monthly to discuss waiting times…I come as a referral so if someone wants to come to our scheme…I can get in immediate contact with them and put a face to the name”.*

This is a clear example of how this referral could work from a logistical perspective. Involvement from an early stage can help allay any fears the cancer patient may have about joining a programme, because “if I’m there at that very moment or at least a phone call away, it’s not actually that bad at all” (P1). Increasing this communication between HCPs, exercise practitioners, and cancer patients is important for programme success, as articulated by P4 who stated that “if you can build a rapport with a hospital and they know you are a reliable trainer who is knowledgeable, the [HCPs] will be much more comfortable prescribing their patients to your programme”. This enhanced communication can help develop trusting, professional relationships between the HCP and exercise practitioner, ensuring that the HCP feels comfortable recommending a cancer patient to a PA programme and continuity of care. It can also ensure that any barriers with regards to a cancer patient’s misconceptions regarding the exercise practitioner’s role and capabilities are addressed, with P3 highlighting that “if you’re…based in a local leisure centre conducting the programme, some patients might associate that with personal trainers that are perhaps under-qualified, inappropriate for them”. Having the HCPs ‘on side’ is important not just for communication, but also as a position of authority who can confirm that the referral is appropriate and will be beneficial, because “if the doctor told a patient to go and exercise, or a practitioner told you to exercise, they are more likely to listen to the doctor…it comes down to authority and status” (P2).

### 3.4. Impact of Policy and Government Support

The exercise practitioners highlighted examples of how support for PA initiatives and HCP referrals could be improved. Much of the provision discussed reflected local pockets of good practice with support from commissioners and funders but also the challenges associated with such delivery. For instance, one participant was from a football community trust and described the work they did:

*“We as a programme have been given funding and [have] currently expanded across the region due to the lack of services that exist across the rest of this area. In terms of this region as a whole, we are the only initiative that provides the service of cancer rehabilitation exercise sessions”.*

(P1)

P1 also described the ongoing challenge of securing funding for the programme, stating that “we have to work incredibly hard for our funding as we have to consistently do the right things that our partners agree with and find a positive influence”. This highlights that metrics of success rely on consistent levels of output being achieved from the funder’s perspective. Persuading the NHS commissioners to invest in rehabilitation programmes was also difficult, as one participant reported:

*“The NHS can’t seem to see that if they took a very small bit out of their budget to fund this, then they would save millions due to discharge times reducing, patients needing more treatments etc. We need to start thinking of exercise as a ‘drug’… to help improve quality of life”.*

(P5)

Exercise practitioners suggested that there was a need for a strategic lead to be taken at a national or governmental level, with P4 highlighting that “initially it needs to be from higher up, so governing bodies get on board and recognise the benefits of the local programmes and what impact they have on patients’ lives”. The interviewees also highlighted a lack of qualified people to support exercise for cancer rehabilitation, and specifically that “you would not feel comfortable sending a recently diagnosed cancer patient down to the local gym with the local personal trainer who is only bothered about getting bigger” (P4). It was suggested that there exists a need for a national approach to upskilling trainers who can work with cancer patients and be given the opportunity to ‘showcase’ their skills to HCPs and thus encourage referrals. Furthermore, exercise practitioners highlighted the absence of PA promotion in cancer care settings. “A lot of them (HCP’s) haven’t had the training and think that they won’t speak about exercise because it takes too much time” (P5). It seemed that PA was often forgotten about, and one of our interviewees suggested a checklist which included discussing PA. “During a patient’s follow-up, the oncologist will be checking off various things to do with the patient’s progress and at the bottom there can be a simple ‘are they safe enough to exercise?’ button and if they select that they are, then an automated email is sent over to admin and they can refer the patient to a local programme... if it becomes successful, then it will have a huge impact on participation levels” (P4). Influencing policy around future oncologist and cancer patient follow-up meetings to involve a discussion of PA would therefore appear to be a recommendation. Finally, an important aspect emerging from the data was the need to appreciate individual preferences along with the need for training and CPD. “The structured programmes are amazing for exercise participation, but for some cancer survivors, they don’t want the constant help and would rather do the exercise themselves, so maybe the gym workers need to have special training for what to look out for when they have a member who is a cancer survivor and can recognise the side effects or if something is wrong” (P4). 

## 4. Discussion

The aim of the current study was to explore the role of PA in cancer patients’ recovery from the perspective of exercise practitioners. The key findings were the importance of social support, whereby cancer patients developed family-like relationships with each other, resulting in increased self-efficacy, and thus encouraged them to continue participation with the PA programme. On the reverse, exercise practitioners felt cancer patients were concerned about PA and the ill effect it may have on them. Finally, a lack of knowledge and resources/training for HCPs were viewed as important and something to improve.

The current study highlighted the importance of social support in terms of the empathic understanding that existed between cancer patients, which was likened to a sense of a family. The findings resonate with Mikkelsen et al. [48], who interviewed 23 lung and pancreatic cancer survivors and found social support to be a key motivating factor for participation in PA, as it provided them with social interaction, enjoyment, and a sense of shared understanding. Social benefits therefore seem to be both a determinant and consequence of PA and their inclusion, and both in the planning and evaluation of cancer rehabilitation programmes is therefore recommended. It should also be noted, however, that not every cancer patient benefitted from a social environment, with some wanting to be undisturbed while engaging in PA. It is important to understand that every cancer patient’s experience of living with cancer will be different, and this reinforces the need for the individualisation of PA, as well as the importance of an exercise practitioner’s role in ensuring that all cancer patients are appropriately accommodated for [23]. Blaney et al. [49] supported this notion, acknowledging that whilst some cancer patients may favour individual, home-based PA, many considered supervised, individualised, group-based sessions to be their preferred form of PA delivery, in order to allow for the sharing of experiences, motivational support, and shared subjective norms. The notion of subjective norms and shared social experiences has been demonstrated in several theories of motivation and behaviour change as facilitators of sustained engagement in a given behaviour; indeed, the theory of planned behaviour [50] has been posited as a viable framework for cancer rehabilitation PA interventions [51]. The current study therefore lends support to the importance of social support as a key facilitator of motivation within cancer rehabilitation exercise programmes.

Patients’ understanding of both physical and psychological benefits of PA emerged as another influential factor with regards to their engagement in the rehabilitation programme. Patients were unaware that PA is considered an appropriate treatment as part of their cancer recovery, which suggests that information regarding its benefits requires improved communication, as previously suggested by James-Martin et al. [52]. The psychological benefits of PA were stated as largely unknown, or unappreciated by the cancer patients; when initially starting the programme, they possessed a ‘fear of failure’ and could not understand how they would be able to take part in PA without the onset of immediate fatigue [34,53]. A lack of knowledge of, and/or belief in, the benefits of PA appeared to undermine their physical engagement, and it is therefore important that HCPs communicate the benefits of PA and lend support to patient engagement. This finding supports previous research by Blaney et al. [53], who found that despite previous experience of PA and therefore its benefits, cancer patients also harboured concerns regarding their levels of fatigue and how this may hinder engagement in PA. It therefore appears common for cancer patients to understand the benefits of PA in healthy individuals, but they lack an awareness of what it can do to help alleviate post-treatment symptoms such as fatigue and weakness and therefore they also lack a belief in their ability to engage. Nonetheless, once patients do engage, they become increasingly aware of the varied and multiple benefits that PA provides, not least of which is an increase in self-esteem and a decrease in anxiety. The cyclical nature of self-efficacy is thus evidenced in cancer patients engaging in PA for cancer rehabilitation, as it can be seen to both encourage and undermine a behaviour. Self-efficacy theory acknowledges how the construct can impact behaviour through cognitive, affective, and motivational mediating processes [54], and HCPs should therefore be cognisant of how each individual cancer patient’s mindset, and therefore level of self-efficacy, may impact their individual behaviour (i.e., engagement) with regards to PA. Exercise practitioners have also suggested that cancer patients tend to set unrealistic goals, whether those be over or under ambitious. This is where skilled HCP input is vital to PA participation for physical and psychological improvements to ensure that the goals set are appropriately planned, achievable, in line with patient needs, and can help increase motivation. Exercise practitioners suggested that setting small weekly goals helps to increase cancer patients’ motivation and alters their defeatist thought processes and attitudes as they begin to realise that they can achieve their goals. Having skilled staff who can implement, and support goal setting is therefore essential [23,55]. The ultimate aim would involve exercise practitioners, HCP’s and cancer patients designing realistic goals together, with the exercise practitioners/HCP’s providing guidance on what they believe is achievable.

A further key finding of the study was the lack of standardised knowledge on the benefits of PA within the HCP population, as well as the lack of motivation to prescribe cancer patients to local PA programmes. Jones, Courneya, Peddle and Mackey [56] found that 55.8% of oncologists viewed PA during treatment as ‘important’, while 29.5% thought their patients were ‘capable of PA’. However, the current study found that the exercise practitioners believe it should be their job to decide whether a cancer patient is capable of PA or not. HCPs were considered to possess varying levels of knowledge of the benefits of PA on cancer patients, which results in associated variability with regard to the extent referrals are made. HCPs were also considered to lack awareness of external PA cancer rehabilitation programmes that are not affiliated with hospitals. Findings from Rutherford et al. [23] support this, whereby they reported that HCPs had varying degrees of awareness of PA programmes such as CARE, which is run externally in the community through funding from a major cancer charity. Daley et al. [57] suggested that when oncologists give advice regarding PA, they do not provide any recommendations on which programmes are best to join in the local area. Furthermore, it has been suggested that cancer patients learn of such PA programmes through media sources rather than from an HCP [58], and that 15% of doctors did not possess adequate knowledge of these referral schemes, nor were they aware of their aims [59]. However, the issue may not lay with the HCPs but may be due to a lack of appropriate opportunities for the HCPs to gain knowledge of PA referral options for their cancer patients. These are real concerns, especially in light of the recent CARE initiative which highlighted that individuals living with cancer could adopt and maintain PA levels in a safe and supportive environment, enabling them to positively impact their physical (fatigue) and psychological (quality of life) function [23]. The effectiveness of certain recent PA programmes helps to further highlight a knowledge gap and support the notion of specific roles, the importance of adopting implementation and behavioural science approaches, whilst considering sport science input/buy-in to increase uptake and remove a potential burden from HCPs. This engagement/buy-in is imperative, especially as the benefits of PA are known; therefore, behavioural changes are required by cancer patients, HCPs and potentially sport scientists to support this. Graham et al. [59] interviewed oncologists who stated that there is no systematic approach for referring patients to external PA programmes, and that it would only be considered if the patients asked them to. Based on this current study, this does not seem to have changed, and further research is required to explore effective ways to improve referral pathways for cancer patients (Table 1). 

The current study identified a dearth of training resources given to HCPs for understanding the benefits of PA, leading to reduced referral rates, as the possibility is often discredited as a viable post-treatment plan. The lack of a standardised approach can lead to a lack of funding for the external PA programmes due to the low number of referrals. With a service that is under resourced and underfunded, programmes cannot operate to their full potential, and ultimately this contributes to the distinct shortage of PA programmes for cancer rehabilitation. Exercise practitioners have suggested that governing bodies need to become more involved with the PA programmes to help maintain cancer patients’ health and well-being, as well as reducing the need for hospital admissions. This notion was supported by Stevinson and Fox [60], who found that 76% of oncologists suggested a lack of readily available resources for establishing a specific PA cancer rehabilitation service. Cancer Research UK [61] also recognise a lack of opportunities for training health professionals in the field of cancer in general, not just with regards to the benefits of PA. They stated that there is an ‘increasingly critical shortage of staff with the right skills in our health system’, and a strategic approach with sustained funding is therefore required to help train staff to ensure that long term outcomes of cancer survival rates can be achieved and that the broader health, wellbeing, and social impacts are realised. This would allow knowledge to be shared and training to be undertaken, positively impacting the HCPs capacity to support cancer patients with their recovery process and using PA to achieve this.

Limitations of this study include a small sample size, which is partly due to there being a lack of cancer rehabilitation exercise practitioners who lead PA sessions. Nonetheless, the study captured the in-depth views of exercise practitioners which are, for the most part, neglected in the current literature and therefore provide a unique insight into beliefs related to PA and cancer patients and the challenges associated with delivering this as a treatment.

## 5. Conclusions

The present study highlights the importance of a supportive social network/environment for cancer patients to inspire one another and have a reassuring figure for when they are feeling defeated; this support network is imperative and must be considered in the future design and development of interventions. Neither cancer patients nor HCP’s possess a complete understanding of the benefits of PA on cancer recovery, and this needs to be improved to ensure that participation levels in PA programmes increases. One of the main causes of insufficiencies in PA knowledge was a lack of funding. The restrictions on funding within this area means that there are a limited number of community-based PA initiatives and cancer patients are not aware of their existence. Funding also may restrict HCPs, as there is no standardised training on the benefits of PA for cancer recovery, leading to discrepancies within their knowledge and negatively affecting referral rates to external PA programmes. To help ensure the smooth referral to programmes, exercise practitioners felt that HCP’s must ensure that their departments are united in understanding PA as a standard of care and truly appreciate its benefits on cancer recovery and improving broad patient outcomes.

## Figures and Tables

**Table 1:** Cancer Rehabilitation Exercise Practitioners Perspectives for Key Design Considerations for Future Programmes.

Social Support
Tailoring to cancer patients needs
Training of staff around effective PA for cancer patients
Promotion with HCPs using multiple approaches such as external opportunities Strategic approach

PA = Physical activity; HCPs = Health Care Practitioners.

## Data Availability

The data presented in this study and the interview schedule are available on request from the corresponding author. The data are not publicly available due to ethical and GDPR reasons.
